# MicroRNA‐20a‐5p contributes to hepatic glycogen synthesis through targeting p63 to regulate p53 and PTEN expression

**DOI:** 10.1111/jcmm.12835

**Published:** 2016-03-28

**Authors:** Weiwei Fang, Jun Guo, Yuan Cao, Shuyue Wang, Cheng Pang, Meng Li, Lin Dou, Yong Man, Xiuqing Huang, Tao Shen, Jian Li

**Affiliations:** ^1^ Graduate School of Peking Union Medical CollegeChinese Academy of Medical SciencesBeijingChina; ^2^ The Key Laboratory of GeriatricsBeijing Institute of Geriatrics and Beijing HospitalMinistry of HealthBeijingChina

**Keywords:** miR‐20a‐5p, glycogen synthesis, p63, p53, PTEN

## Abstract

Recently, it is implicated that aberrant expression of microRNAs (miRs) is associated with insulin resistance. However, the role of miR‐17 family in hepatic insulin resistance and its underlying mechanisms remain unknown. In this study, we provided mechanistic insight into the effects of miR‐20a‐5p, a member of miR‐17 family, on the regulation of AKT/GSK pathway and glycogenesis in hepatocytes. MiR‐20a‐5p was down‐regulated in the liver of db/db mice, and NCTC1469 cells and Hep1‐6 cells treated with high glucose, accompanied by reduced glycogen content and impaired insulin signalling. Notably, inhibition of miR‐20a‐5p significantly reduced glycogen synthesis and AKT/GSK activation, whereas overexpression of miR‐20a‐5p led to elevated glycogenesis and activated AKT/GSK signalling pathway. In addition, miR‐20a‐5p mimic could reverse high glucose‐induced impaired glycogenesis and AKT/GSK activation in NCTC1469 and Hep1‐6 cells. P63 was identified as a target of miR‐20a‐5p by bioinformatics analysis and luciferase reporter assay. Knockdown of p63 in the NCTC1469 cells and the Hep1‐6 cells by transfecting with siRNA targeting p63 could increase glycogen content and reverse miR‐20a‐5p inhibition‐induced reduced glycogenesis and activation of AKT and GSK, suggesting that p63 participated in miR‐20a‐5p‐mediated glycogenesis in hepatocytes. Moreover, our results indicate that p63 might directly bind to p53, thereby regulating PTEN expression and in turn participating in glycogenesis. In conclusion, we found novel evidence suggesting that as a member of miR‐17 family, miR‐20a‐5p contributes to hepatic glycogen synthesis through targeting p63 to regulate p53 and PTEN expression.

## Introduction

MicroRNAs (miRs), a group of small (approximately 22nt) non‐coding RNA molecules which are found extensively in plants, animals and some viruses, have been confirmed to participate in diverse cellular phenotypes in the form of mRNA‐miRNA interactions 1. MiRs can directly bind a group of mRNAs by pairing with their 3′untranslated regions (UTRs) 2. Besides, miRs can be regulated by long non‐coding RNA (lncRNA) or circular RNA (circRNA), consequently, directing diseases development 3. Recently, numerous investigations have implicated that aberrant expression of miRs is associated with insulin resistance. For example, miR‐29 was highly up‐regulated in diabetic rats and overexpression of miR‐29 in 3T3‐L1 adipocytes resulted in insulin resistance 4. It was reported the central importance of miR‐103/107 to insulin sensitivity by regulating cavolin‐1 expression 5. Anti‐miR‐320 oligo was found to regulate insulin resistance in adipocytes by improving PI3‐K pathway 6. In addition, miR‐320 participated in insulin resistance in adipocytes through targeting p85, a kinase subunit of PI3K 6. Moreover, up‐regulation of miR‐126 in hepatocytes led to a substantial reduction in IRS‐1 protein expression, and a consequent impairment in insulin signalling 7. Recently, Yang *et al*. unveiled a novel mechanism whereby miR‐15b is linked causally to the pathogenesis of hepatic insulin resistance in saturated fatty acid‐induced obesity 8. In a previous study, we found that miR‐200s and miR‐301a contributed to interleukin (IL)‐6‐induced hepatic insulin resistance 9, 10. Furthermore, we indicated that miR‐19a regulated PTEN expression to mediate glycogen synthesis in hepatocytes 11.

The miR‐17/20 cluster was identified as a tumour suppressor in human breast cancer by reducing the expression of AIB1 and cycling D1 12, 13. Akt1 was needed to induce breast cancer cells apoptosis by miR17/20 14. MiR‐17 family consists of six distinct mature miRs, including miR‐20a‐5p, miR‐20b‐5p, miR‐106a‐5p, miR‐106b‐5p, miR‐17‐5p and miR‐93‐5p which possess the same ‘seed sequence’ (AAAGUG). In addition, the members of miR‐17 family were implicated in myelodysplastic syndromes, Cisplatin‐Resistant and Metastasis, Lesch–Nyhan syndrome by targeting different associated genes 15, 16, 17, 18. However, the role of miR‐17 family in hepatic insulin resistance and its underlying mechanisms remain unknown. In the present study, we found novel evidence suggesting that as a member of the miR‐17 family, miR‐20a‐5p might contribute to hepatic glycogen synthesis through targeting p63 to regulate p53 and PTEN expression.

## Materials and methods

### Human liver specimens

The human liver biopsies were performed with patient consent within the diagnostic workup of NAFLD. The application of patient‐derived materials was approved by the Research Ethics Committee of Beijing You‐An Hospital, and written consent was obtained from all patients.

### Cell culture

NCTC1469, a murine liver cell line, was cultured in DMEM supplemented with 10% (v/v) Horse serum (Hyclone, Logan, UT, USA), 80 units/ml penicillin and 80 μg/ml streptomycin (Life Technologies, Inc., Carlsbad, CA, USA), at 37°C in a humidified atmosphere with 5% CO_2_.

Hep1‐6, a cell line from mouse hepatoma, was cultured in DMEM containing 10% (v/v) foetal bovine serum, 80 units/ml penicillin and 80 μg/ml streptomycin, at 37°C in a humidified atmosphere with 5% CO_2_.

### Transient transfection

Firstly, 6 × 10^5^ cells were equally seeded in the 6‐well plates with 2 ml DMEM culture medium containing serum and antibiotics. At the same time, miR‐20a‐5p mimic, inhibitor or miR‐negative control (Genepharma, Shanghai, China) were mixed with HiperFect transfection reagent (Qiagen, Duesseldorf, Germany) and incubated at room temperature for 10 min. The complex was then respectively transfected into NCTC1469 cells and Hep1‐6 cells for 48 hrs.

### RNA extraction and real‐time PCR

The total RNA from NCTC1469 cells and Hep1‐6 cells was extracted with Trizol (Invitrogen, Carlsbad, CA, USA) rigorously according to the manufacturer's instructions. The concentration and the purity of the RNA samples were assayed by absorbent density analysis on OD260/OD280.

To get cDNA sequence of the specific miR, 2 μg of the total RNA was reversely transcribed using Taq‐Man MicroRNA Reverse Transcription Kit (Applied Biosystems, Carlsbad, CA, USA) with specific primers for miR‐20a‐5p and U6 (Shanghai Sangon Technology, Shanghai, China). To quantify the miR‐20a‐5p, a quantitative real‐time PCR assay was performed with SYBR Green Supermix (Bio‐Rad, Hercules, CA, USA) in a BIO‐RAD iCycleriQ real‐time PCR detection system. The PCR amplifications were performed in a 10‐μl reaction system containing 5 μl SYBR Green Supermix, 0.4 μl forward primer, 0.4 μl reverse primer, 2.2 μl ddH_2_O and 2 μl template cDNA. The thermal cycling conditions were a hot start step at 95°C for 10 min., followed by 40 cycles at 95°C for 15 sec. and 60°C for 1 min. The relative level of miR‐20a‐5p was determined using the 2‐delta delta Ct analysis method. We choose U6 as the endogenous control. Nucleotide primers used for reverse transcription were as follows (5′‐3′): miR‐20a‐5p, GTCGTATCCAGTGCAGGGTCCGAGGTATTCGCACTGGATACGACCTACC; U6, GTCGTATCCAGTGCAGGGTCCGAGGTATTCGCACTGGATACGACAAATATG.

The primers used for real‐time PCR were as follows (5′‐3′): miR‐20a‐5p forward, GCGCTAAAGTGCTTATAGTGCA; U6 forward, GCGCGTCGTGAAGCGTTC; Universal reverse primer, GTGCAGGGTCCGAGGT.

### Protein extraction and Western blot analysis

Proteins were extracted from the NCTC1469 cells and hep1‐6 cells in RIPA buffer (1% TritonX‐100, 15 mmol/l NaCl, 5 mmol/l ethylenediaminetetraacetic acid, and 10 mmol/l Tris‐HCl, pH 7.0) (Solarbio, Beijing, China) supplemented with a protease and phosphatase inhibitor cocktail (Sigma‐Aldrich, St. Louis, MO, USA). The mixed protein from the cell lysates were separated by 10% SDS‐PAGE and transferred electrophoretically to a polyvinylidene fluoride (PVDF) membrane. After soaking with 8% milk in PBST (pH 7.5) for 2 hrs at room temperature, the membranes were incubated with the following specific primary antibodies: anti‐p63, anti‐p53, anti‐PTEN, anti‐p‐AKT (ser473), anti‐p‐GSK (ser9), anti‐AKT, anti‐GSK and anti‐GAPDH (Santa Cruz Biotechnology, Inc., Dallas, TX, USA). After incubating for 24 hrs, the corresponding HRP‐conjugated anti‐rabbit or mouse IgG secondary antibodies (all at a 1:5000; Zhongshan gold bridg, Inc., Beijing, China) were subsequently applied and immunodetection was achieved using the ECL plus detection system (Millipore, Boston, MA, USA) according to the manufacturer's instructions. The house‐keeping gene GAPDH was used as the internal 1ontrol.

### Immunofluorescence

NCTC1469 cells were cultured on 6‐well plates with glass coverslips and fix the samples in 4% paraformaldehyde for 30 min. at room temperature. The samples were washed three times in PBS for 5 min. per time. Then, the coverslips were incubated with the diluted antibody against p63, p53 and PTEN (1:50 diluted in PBS) in a humidified chamber overnight at 4°C. After washing with PBS for three times (5 min. per time), the slides were incubated accordingly with TRITC‐conjugated anti‐rabbit or mouse IgG (1:500 diluted in PBS) mixed with DAPI (1:1000 diluted in PBS) for 20 min. at room temperature. After decanting the secondary antibody solution and washing three times with PBS in dark, coverslips were mounted with a drop of mounting medium and coated to glass slides. The slides were sealed at room temperature for about 1 hr in dark. The fluctuation of fluorescence intensity was then examined using a fluorescence microscope.

### Glycogen content measurement

Glycogen levels were measured in cells or liver tissues incubated for 3 hrs in the presence of 1 nmol/l insulin (Usbio, Swampscott, MA, USA), using a glycogen assay kit (Biovision, San Francisco, CA, USA).

### Luciferase target assay

The 3′UTR of p63 containing the predicted target site for miR‐20a‐5p were cloned into the pmirGLO (Promega, Madison, WI, USA) luciferase reporter vector which has been cleavage at SacI and XhoI sites. Details of PCR procedures are described as follows: a hot start step at 95°C for 10 min., followed by 40 cycles at 95°C for 15 sec. and 55°C for 45 sec., 72°C for 30 sec. The mutant was cloned using the Fast Mutagenesis System (TransGen Biotech, Beijing, China).

Before conducting luciferase reporter assay, 5 × 10^4^ cells per well were seeded in 24‐well plates in a 500‐μl medium and cultured for 18 hrs. The cells were transfected with the modified firefly luciferase vector (500 ng/μl) mixed with Vigofect transfection Reagent strictly according to the manufacturer's instruction. After continuous exposure for 48 hrs, the luciferase activities from firefly and renilla were measured with the Dual‐luciferase reporter assay system (Promega). We used renilla activity as the normalized parameter.

### Inhibition of p63 and p53 expression by RNA interference

Before transfection, 1 × 10^5^ cells per well were seeded in a 6‐well plate for application. The siRNA targeting p63/p53 or negative control which was purchased from Genepharma was transfected into cells for 48 hrs using HiperFect transfection reagent (Qiagen) as described above. The siRNA sequences are listed as follows: sip63‐1 sense: 5′‐GGAAUGAACAGACGUCCAATT‐3′; sip63‐2 sense: 5′‐GCUGAGCCGUGAGUUCAAUTT‐3′; sip53 sense: 5′‐AAGUCUGUUAUGUGCACGUACTT‐3′.

### Statistical analysis

Data were presented as mean ± S.D. from three independent experiments or five mice. Statistical analysis was carried out with Student's *t*‐test. *P* < 0.05 was considered as statistically significant difference.

## Results

### Down‐regulation of miR‐20a‐5p is accompanied by reduced glycogen synthesis

**Figure 1:**
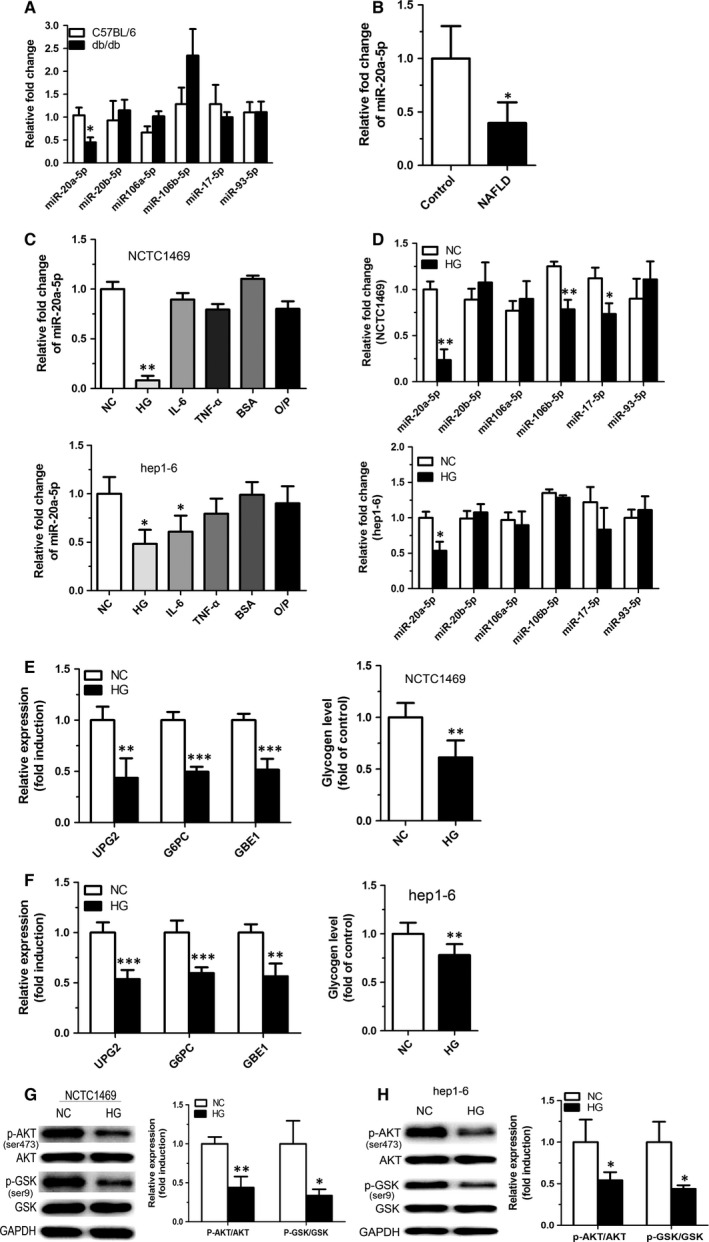
Down‐regulation of miR‐20a‐5p is accompanied by reduced glycogen synthesis. The levels of miR‐20a‐5p and other five members of miR‐17 family including miR‐20b‐5p, miR‐106a‐5p, miR‐106b‐5p, miR‐17‐5p and miR‐93‐5p were measured in the liver of db/db mice (**A**). The level of miR‐20a‐5p was detected in patients with NAFLD (**B**). Murine NCTC 1469 and Hep1‐6 hepatocytes were stimulated with 33.3 mmol/l glucose for 48 hrs, 0.25 mmol/l palmitate for 24 hrs, 10 nmol/l IL‐6 or 10 nmol/l TNF‐α for 24 hrs respectively. The level of miR‐20a‐5p was determined in both cells (**C**). The changes of miR‐17 family members were detected in NCTC 1469 and Hep1‐6 cells stimulated with 33.3 mmol/l glucose for 48 hrs (**D**). Genes related to hepatic glycogen synthesis, glycogen level (**E** and **F**) and activation of AKT and GSK (**G** and **H**) were analysed in NCTC 1469 and Hep1‐6cells treated with 33.3 mmol/l glucose for 48 hrs. Data represent the mean ± S.D. *N* = 5 mice or *N* = 3 independent experiments. *P < 0.05; **P < 0.01 (*versus* control).

In previous study, hepatic miR profiles of db/db mice (a diabetes model) and C57/BL mice were analysed by miR microarray. The results showed decreased expression of miR‐20a‐5p in the liver of db/db mice compared in that of C57/BL mice. To confirm the chip results, the changes of miR‐20a‐5p and other five members of miR‐17 family including miR‐20b‐5p, miR‐106a‐5p, miRNA‐106b‐5p, miR‐17‐5p and miR‐93‐5p were examined using real‐time PCR. As shown in Figure 1A, miR‐20a‐5p, but not miR‐20b‐5p, miR‐106a‐5p, miR‐106b‐5p, miR‐17‐5p and miR‐93‐5p, was significantly down‐regulated. The expression of miR‐20a‐5p significantly decreased as well in human with NAFLD combined with insulin resistance (Fig. 1B). The clinical and biochemical characteristics of the healthy controls and NAFLD patients are demonstrated in Table 1. The age and gender distribution were similar in both groups. The characteristics including BMI, waist circumference and triglyceride level were obviously higher in the NAFLD patients. To further analyse the changes of miR‐20a‐5p in a cellular model of insulin resistance, murine NCTC 1469 and Hep1‐6 hepatocytes were stimulated with 33.3 mmol/l glucose for 48 hrs, 0.25 mmol/l palmitate for 24 hrs, 10 nmol/l IL‐6 or 10 nmol/l tumour necrosis factor (TNF)‐α for 24 hrs respectively. The results showed that glucose, but not palmitate, IL‐6 and TNF‐α, could down‐regulate miR‐20a‐5p expression in both cells (Fig. 1C). To confirm the connection between miR‐20a‐5p and high glucose, the changes of miR‐20a‐5p and other five members of miR‐17 family including miR‐20b‐5p, miR‐106a‐5p, miRNA‐106b‐5p, miR‐17‐5p and miR‐93‐5p were analysed in murine NCTC 1469 and Hep1‐6 hepatocyte stimulated with 33.3 mmol/l glucose for 48 hrs (Fig. 1D). Moreover, high‐glucose treatment led to decreased expression of genes (UPG2, G6PC and GBE1) related to hepatic glycogen synthesis, impaired glycogen synthesis (Fig. 1E and F) and activation of AKT and GSK (Fig. 1G and H). These data indicated that down‐regulation of miR‐20a‐5p is accompanied by reduced glycogen synthesis.

**Table 1:** Clinical and biochemical characteristics of healthy controls and patients with nonalcoholic fatty liver disease1 *n* (%)

Characteristic	Control (*n* = 10)	NAFLD (*n* = 10)	*P* value
Gender (males/females)	5/5	5/5	–
Age (year)	43.8 ± 9.4	44.6 ± 9.0	0.847736
BMI (kg/m^2^)	22.5 ± 3.2	27.0 ± 3.0	0.004389
Smoking	No	No	–
Waist circumference (cm)	77.4 ± 9.6	93.1 ± 8.6	0.001175
Diabetes mellitus	No	No	–
Metabolic syndrome	No	No	–
Hypertension	No	2 (20)	–
Systolic blood pressure (mmHg)	110.6 ± 9.6	114.4 ± 7.5	0.336856
Diastolic blood pressure (mmHg)	73.4 ± 7.2	77.5 ± 5.1	0.159605
AST median (min‐max, U/l)	15.5 (14–24)	18.5 (12–26)	0.460403
ALT median(min‐max, U/l)	11 (6–22)	18.5 (12–26)	0.460402
Total cholesterol (mmol/l)	4.6 ± 0.5	5.2 ± 1.0	0.131255
HDL‐cholesterol (mmol/l)	1.5 ± 0.3	1.3 ± 0.2	0.045348
LDL‐cholesterol (mmol/l)	2.6 ± 0.4	2.9 ± 1.3	0.477027
Triglycerides (mmol/l)	0.9 ± 0.3	2.5 ± 1.6	0.005397

### MiR‐20a‐5p contributes to glycogenesis in hepatocytes

**Figure 2:**
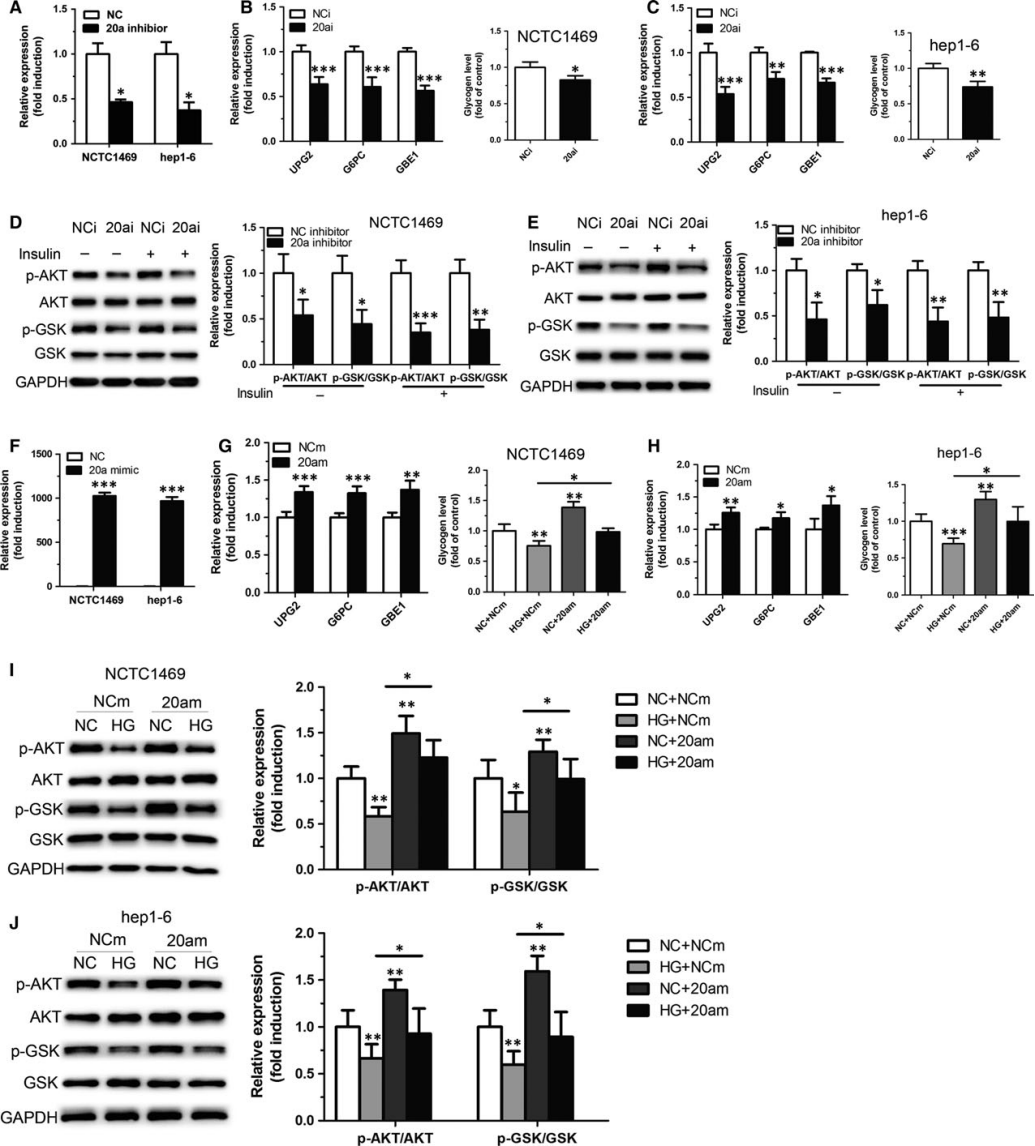
MiR‐20a‐5p contributes to glycogenesis in hepatocytes. The levels of miR‐20a‐5p (**A**), glycogen synthesis (**B** and **C**) and phosphorylation of AKT and GSK (**D** and **E**) were analysed in NCTC1469 cells and Hep1‐6 cells transfected with miR‐20a‐5p inhibitor. The transfected efficiency of miR‐20a‐5p mimic was measured in NCTC1469 cells and Hep1‐6 cells (**F**). Moreover, miR‐20a‐5p mimic was transfected in NCTC1469 cells and Hep1‐6 cells treated with 33.3 mmol/l glucose for 48 hrs. The levels of glycogen synthesis (**G** and **H**) and phosphorylation of AKT and GSK (**I** and **J**) were measured. Data represent the mean ± S.D. *N* = 3 independent experiments. *P < 0.05; **P < 0.01;***P < 0.001 (*versus* control).

To investigate the role of miR‐20a‐5p in glycogen synthesis, miR‐20a‐5p inhibitor was transfected into NCTC1469 cells and Hep1‐6 cells. As shown in Figure 2A, miR‐20a‐5p was down‐regulated by nearly 53% in both cells transfected by miR‐20a‐5p inhibitor. Inhibition of miR‐20a‐5p in NCTC1469 cells and Hep1‐6 cells led to decreased genes expression related to hepatic glycogen synthesis, reduced glycogen synthesis (Fig. 2B and C). Considering that insulin can active the insulin signalling, as assessed by enhanced phosphorylation of AKT and GSK, we analysed the effect of miR‐20a‐5p on the phosphorylation of AKT and GSK under insulin stimulation. As shown in Figure 2D and E, inhibition of miR‐20a‐5p impaired phosphorylation of AKT and GSK. As shown in Figure 2F, miR‐20a‐5p was significantly up‐regulated in both cells. Moreover, miR‐20a‐5p mimic was transfected in NCTC1469 cells and Hep1‐6 cells treated with 33.3 mmol/l glucose for 48 hrs. Overexpression of miR‐20a‐5p significantly reversed high glucose‐induced reduced glycogenesis (Fig. 2G and H) and impaired AKT/GSK activation in NCTC1469 and Hep1‐6 cells (Fig. 2I and J).

### P63 is identified as a target of miR‐20a‐5p

**Figure 3:**
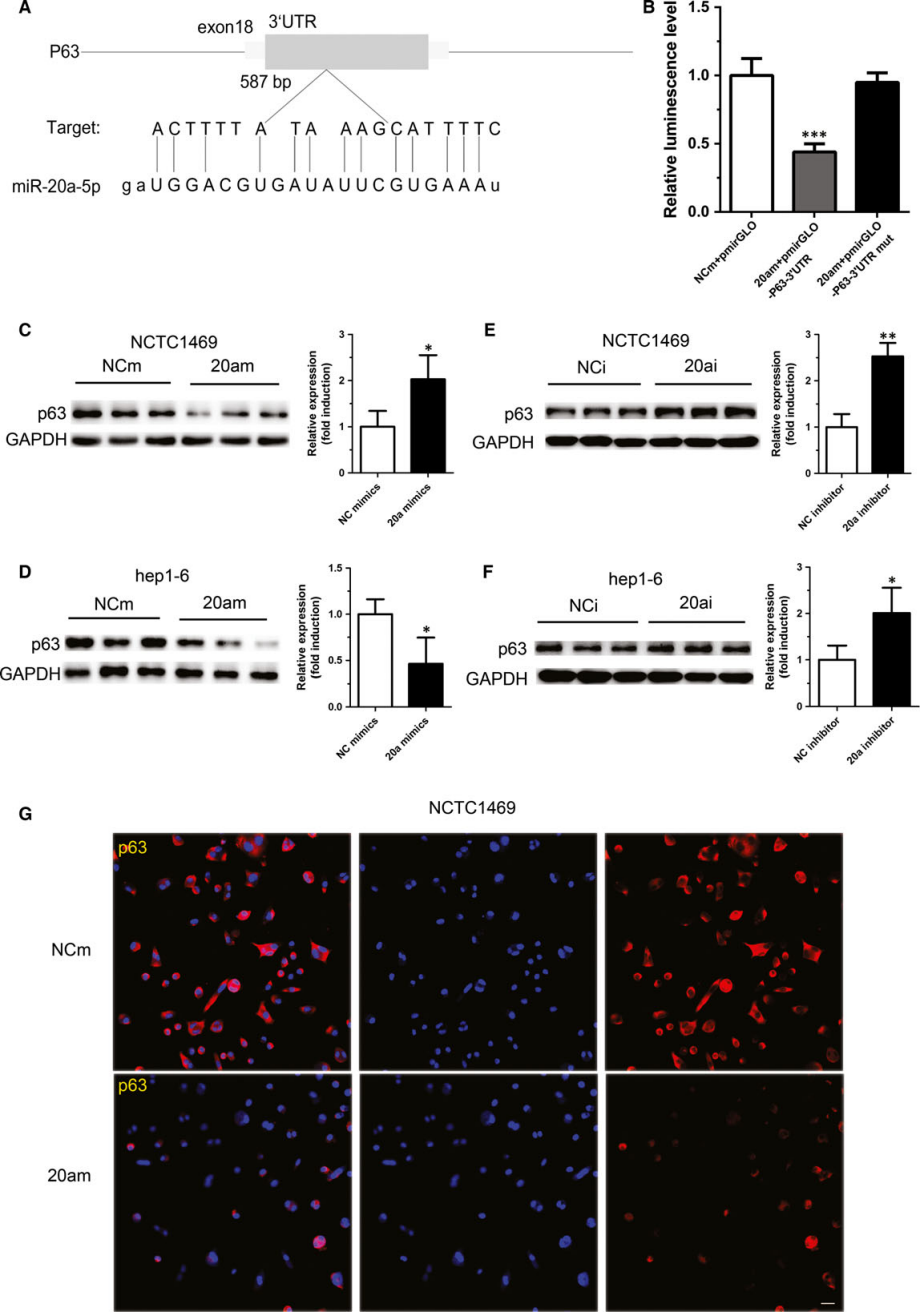
P63 is identified as a target of miR‐20a‐5p. A binding site of miR‐20a‐5p on the 3′UTR of p63 was analysed by TargetScan (**A**). The 3′UTR of p63 containing the predicted binding site was cloned into pGLOmiR vector. The luciferase activity in the 293T cells transfected with pmirGLO‐p63‐3′UTR or pmirGLO‐p63‐3′UTR mutant and miR‐20a‐5p mimic was tested by Dual‐luciferase reporter assay (**B**). The p63 expression was measured in NCTC146 cells and Hep1‐6 cells transfected with miR‐20a‐5p mimic (**C** and **D**) or miR‐20a‐5p inhibitor (**E** and **F**). In addition, the fluorescence intensity of p63 was attenuated in the NCTC1469 cells treated with miR‐20a‐5p mimic for 48 hrs, as shown by immunofluorescence assay (**G**). Scale bar represents 20 μm. Data represent the mean ± S.D. *N* = 3 independent experiments. *P < 0.05; **P < 0.01(*versus* control).

We next sought to identify the potential target of miR‐20a‐5p involving in glycogen synthesis through computational miRNA target prediction databases. Analysis from TargetScan revealed that there is a binding site of miR‐20a‐5p on the 3′UTR of p63 (Fig. 3A). The 3′UTR of p63 containing the predicted binding site was then cloned into pGLOmiR vector. Dual‐luciferase reporter assay showed that overexpression of miR‐20a‐5p could significantly reduce the luciferase activity in the 293T cells transfected with pmirGLO‐p63‐3′UTR, indicating that p63 is a target of miR‐20a‐5p (Fig. 3B). Moreover, up‐regulation of miR‐20a‐5p significantly suppressed p63 expression in NCTC1469 cells and Hep1‐6 cells (Fig. 3C and D), while inhibition of miR‐20a‐5p obviously elevated the protein level of p63 (Fig. 3E and F). In addition, immunofluorescence assay showed that the fluorescence intensity of p63 was attenuated in the NCTC1469 cells treated with miRNA‐20a‐5p mimic for 48 hrs (Fig. 3G). Together, these data suggest that p63 is a target of miRNA‐20a‐5p.

### Knockdown of p63 improves glycogen synthesis

**Figure 4:**
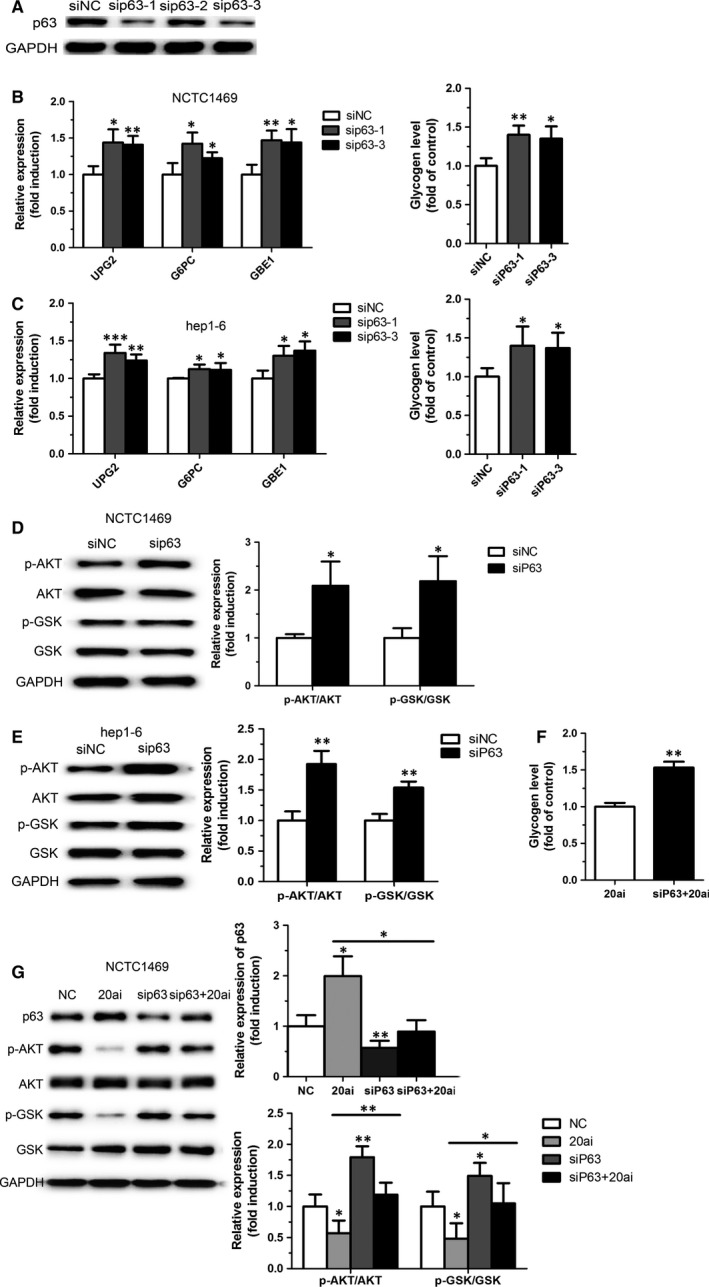
Knockdown of p63 improves glycogen synthesis. Two specific siRNA targeting p63 was selected (**A**). Genes related to hepatic glycogen synthesis, glycogen content (**B** and **C**) and phosphorylation levels of AKT and GSK (**D** and **E**) were measured in the NCTC1469 cells and the Hep1‐6 cells transfected with siRNA targeting p63. Moreover, p63 knockdown could reverse miR‐20a‐5p inhibition‐induced reduced glycogenesis and activation of AKT and GSK (**F** and **G**). Data represent the mean ± S.D. *N* = 3 independent experiments. *P < 0.05; **P < 0.01(*versus* control).

To explore the role of p63 in glycogenesis, two specific siRNAs targeting p63 (sip63‐1 and sip63‐3) were selected (Fig. 4A). As shown in Figure 4B and C, knockdown of p63 increased the expression of genes related to hepatic glycogen synthesis and glycogen content in the NCTC1469 cells and the Hep1‐6 cells transfected with siRNA targeting p63. Moreover, inhibition of p63 expression in NCTC1469 cells and Hep1‐6 cells led to enhanced phosphorylation levels of AKT and GSK (Fig. 4D and E). Most importantly, p63 knockdown could reverse miR‐20a‐5p inhibition‐induced reduced glycogenesis and activation of AKT and GSK (Fig. 4F and G), suggesting that p63 participated in miR‐20a‐5p‐mediated glycogenesis in hepatocytes.

### P63 regulates expression of PTEN by directly binding to p53

**Figure 5:**
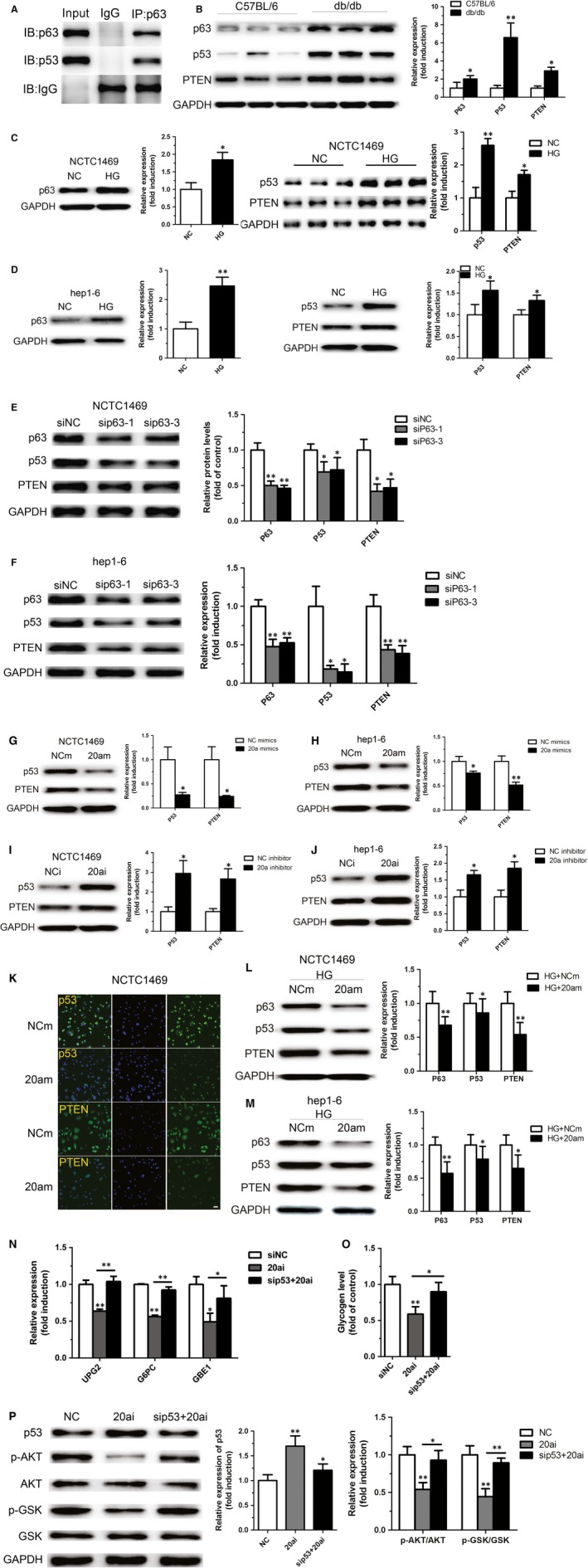
P63 regulates expression of PTEN by directly binding to p53. A direct interaction between p63 and p53 was observed by co‐immunoprecipitation (**A**). The levels of p63, p53 and PTEN protein were measured in the liver of db/db mice (**B**), NCTC1469 cells (**C**) and Hep1‐6 cells (**D**) treated with high glucose as well as NCTC1469 cells (**E**) and Hep1‐6 cells (**F**) transfected with siRNA targeting p63. Overexpression of miR‐20a‐5p led to decreased protein levels of p53 and PTEN (**G** and **H**), whereas inhibition of miR‐20a‐5p increased the expression of p53 and PTEN (**I** and **J**). Transfection of miR‐20a‐5p mimic significantly decreased the fluorescence intensity of p53 and PTEN, as shown by immunofluorescence assay (**K**). Moreover, overexpression of miR‐20a‐5p in NCTC1469 cells and Hep1‐6 cells could reverse high glucose‐induced increased expression of p63, p53 and PTEN (**L** and **M**). Moreover, p53 knockdown could reverse miR‐20a‐5p inhibition‐induced reduced expression of genes related to hepatic glycogen synthesis such as UPG2, G6PC and GBE1 (**N**) and hepatic glycogen synthesis (**O**), and activation of AKT and GSK (**P**) in the NCTC1469 cells treated by miR‐20a‐5p inhibition. Scale bar represents 20 μm. Data represent the mean ± S.D. *N* = 5 mice or *N* = 3 independent experiments. *P < 0.05; **P < 0.01 (*versus* control).

To gain further insights into the mechanisms by which p63 participated in miR‐20a‐5p‐mediated glycogenesis in hepatocytes, we analysed the possible downstream proteins of p63. It was reported that a wide interaction appeared between the members of p53 family 19. As a key member of p53 family, whether p63 could directly bind to p53 has not been validated. Thus, we observed a direct interaction between p63 and p53 by co‐immunoprecipitation. The result showed that p63 could directly bind to p53 (Fig. 5A). Moreover, as shown in Figure 5B, up‐regulation of p63 was accompanied by increased levels of p53 and PTEN protein in the liver of db/db mice. Similarly, NCTC1469 and Hep1‐6 cells treated with high glucose displayed up‐regulation of p63, p53 and PTEN expression (Fig. 5C and D). Importantly, the protein levels of p53 and PTEN were significantly decreased in the NCTC1469 and Hep1‐6 cells transfected with siRNA targeting p63 (Fig. 5E and F). As expect, overexpression of miR‐20a‐5p led to decreased protein levels of p53 and PTEN (Fig. 5G and H), whereas inhibition of miR‐20a‐5p increased the expression of p53 and PTEN (Fig. 5I and J). Immunofluorescence assay showed that transfection of miR‐20a‐5p mimic significantly decreased the fluorescence intensity of p53 and PTEN (Fig. 5K). Notably, overexpression of miR‐20a‐5p in NCTC1469 cells and Hep1‐6 cells could reverse high glucose‐induced increased expression of p63, p53 and PTEN (Fig. 5L and M). Most importantly, as shown in Figure 5N–P, p53 knockdown could reverse miR‐20a‐5p inhibition‐induced reduced expression of genes related to hepatic glycogen synthesis such as UPG2, G6PC and GBE1 and hepatic glycogen synthesis, and activation of AKT and GSK in the NCTC1469 cells treated by miR‐20a‐5p inhibition. These results suggest that p63 might directly bind to p53, thereby regulating PTEN expression and in turn participating in glycogenesis.

## Discussion

As a miR‐17 family member, up‐regulation of miR‐20a‐5p was associated with advanced clinical stages of astrocytoma, indicating the potential of serum miR as the novel diagnostic and prognostic biomarkers for human astrocytoma 20. Comparing Sprague–Dawley with low and Wistar rats with high 11beta‐HSD2 activity revealed miR‐20a‐5p to be differentially expressed, suggesting that miR‐dependent mechanism seems to modulate 11beta‐HSD2 dosage in health and disease states 21. The fact that miR‐20a‐5p was significantly overexpressed in the HBV‐positive HCC patients compared with the HBV‐positive cancer‐free controls in both the training and validation sets indicated miR‐20a‐5p as a blood‐based early detection biomarker for HCC screening 22. MiR‐20a‐5p that was differentially expressed in gestational diabetes mellitus could serve as one of the non‐invasive biomarkers 23. In this study, down‐regulation of miR‐20a‐5p was found in the liver of db/db mice, and NCTC1469 cells and Hep1‐6 cells treated with high glucose, accompanied by reduced glycogen content and impaired insulin signalling. These data suggest that miR‐20a‐5p might be involved in high glucose‐induced hepatic insulin resistance.

Next, we sought to investigate the potential role of miR‐20a‐5p in hepatic insulin resistance. It was reported that miR‐20a‐5p functions as a central hub targeting about 500 genes, many of which play a role in signalling and T‐cell activation, and have transcription factor activity 24. MiR‐20a‐5p could alter cell proliferation and differentiation by down‐regulating the mitogen‐activated protein kinase (MAPK) signalling pathway 25. In addition, miR‐20a‐5p participated in autophagic process by targeting ATG16L1 mRNA, which might be a critical adapting mechanism for ischaemic kidney injury 26. Moreover, miR‐20a‐5p was involved in many types of tumours, such as gastric cancer, colon cancer, breast tumours and nasopharyngeal carcinoma 25, 27, 28, 29. However, the potential role of miR‐20a‐5p in hepatic insulin resistance remains unknown. In the present study, we suggested that miR‐20a‐5p contributes to glycogenesis in hepatocytes. Inhibition of miR‐20a‐5p significantly reduced glycogen synthesis and AKT/GSK activation, while overexpression of miR‐20a‐5p led to elevated glycogenesis and activated AKT/GSK signalling pathway. Notably, miR‐20a‐5p mimic could reverse high glucose‐induced impaired glycogenesis and AKT/GSK activation in NCTC1469 and Hep1‐6 cells.

We further identified the target genes of miR‐20a‐5p. Based on bioinformatics analysis, we found that p63 is a potential target for miR‐20a‐5p. Luciferase reporter assay revealed that miR‐20a‐5p could directly bind to the 3′UTR of p63, which then further inhibit its translation. Western blot analysis showed that overexpression of miR‐20a‐5p inhibited the expression of protein p63, while inhibition of miR‐20a‐5p elevated the protein level of p63. These data suggest that p63 is a target gene of miR‐20a‐5p. P63 is located on chromosome 3q27‐29, and shares three domains that are indispensable for their functions: transactivation domain, DNA binding domain and oligomerization domain 30. P63 protein is implicated in many physiological processes including transformation of induced pluripotent stem cells, oocyte death, non‐small‐cell lung carcinoma, autophagic cell death and epidermal morphogenesis 31, 32, 33, 34. In human keratinocytes, RUNX1 functions as an important transcription factor in the transition from proliferation to differentiation depending on the functional interplay between p63 and p53 controls 35. Fatt *et al*. found that p63 and p73 co‐regulated p53‐dependent neural precursor cell apoptosis *versus* senescence, whereby determining appropriate adult neurogenesis 36. The studies of p63 in more aggressive, metastatic tumours indicated that p63 loss accelerated tumour genesis as well as metastatic spread 37, 38. These researches revealed p63 function as a crucial metastasis suppressor involving an adhesion programme, also emphasized that its interaction with the different isoforms of all p53 family members contributed to the tumour phenotype finally 39, 40, 41. It was proposed that p53 mutants derived by a variety of tumour physically interact with p63 and p73, the members of p53 family, and negatively regulate their proapoptotic function 42. Moreover, the research showed that the deficiency of p63 induced cellular senescence and accelerated ageing phenotypes 43. Recently, the correlation between p63 protein and metabolic disorders is slowly revealed 44. However, despite its wide expression of p63 in various tissues, little study has been done on association between p63 and insulin signalling pathway. In the present study, we found that inhibition of p63 expression in NCTC1469 cells and Hep1‐6 cells by transfecting with siRNA targeting p63 elevated glycogen content and phosphorylation levels of AKT and GSK. Most importantly, p63 knockdown could reverse miR‐20a‐5p inhibition‐induced reduced glycogenesis and activation of AKT and GSK. These data for the first time indicate the adverse effect of p63 on insulin signalling pathway.

As a family member of p53, a common binding domain exists between p63 and p53. It has been revealed that p53 could repress p63 transcription 39, 40. Here, we analysed a direct interaction between p63 and p53 by co‐immunoprecipitation. The result showed that p63 could directly bind to p53. Base on it was reported that p53 could strongly initiate the transcription of PTEN in various cells 45, 46, we proposed that p63 mediated PTEN expression by binding to p53. Chen *et al*. indicated that the cooperative tumour suppression happened between p53 and PTEN in which p53 acts as an important failsafe protein of PTEN‐deficient tumours and acute inactivation of PTEN causes growth arrest through the p53‐dependent cellular senescence pathway no matter *in vitro* or *in vivo* 47. It is reported that PTEN plays a role in maintenance of high p53 acetylation in response to DNA damage and such acetylation promotes p53 tetramerization which is required for the PTEN‐p53 interaction 19. Although these two proteins are functionally distinct, reciprocal interaction and combined action has been confirmed 48. PTEN and p53 were known to regulate each other both at the transcription and protein levels. The PTEN and p53 compound enhances p53 DNA binding and transcriptional activity 49. One way p53 to suppress the expression of PIP3 is by inducing the expression of PTEN indirectly 50. Li *et al*. provided a molecular explanation for their previous observation that PTEN controls p53 protein levels independent of its phosphatase activity 19. In addition, PTEN could reverse chemotherapy resistance mediated by MDM2 by the way of interacting with p53 in Acute Lymphoblastic Leukemia Cells 51. Moreover, the research showed that oroxylin A could promote the stabilization of p53 by PTEN‐mediated negative regulation of MDM2 transcription 45. In the present study, we found that inhibition of p63 could directly inhibit the expression of protein p53 and PTEN, which then activate insulin signal pathway and facilitate the synthesis of glycogen. Importantly, miR‐20a‐5p mimic reduced protein levels of p53 and PTEN, whereas inhibition of miR‐20a‐5p up‐regulated the expression of p53 and PTEN. These results suggest that p63 might directly bind to p53, thereby regulating PTEN expression and in turn participating in glycogenesis.

**Figure 6:**
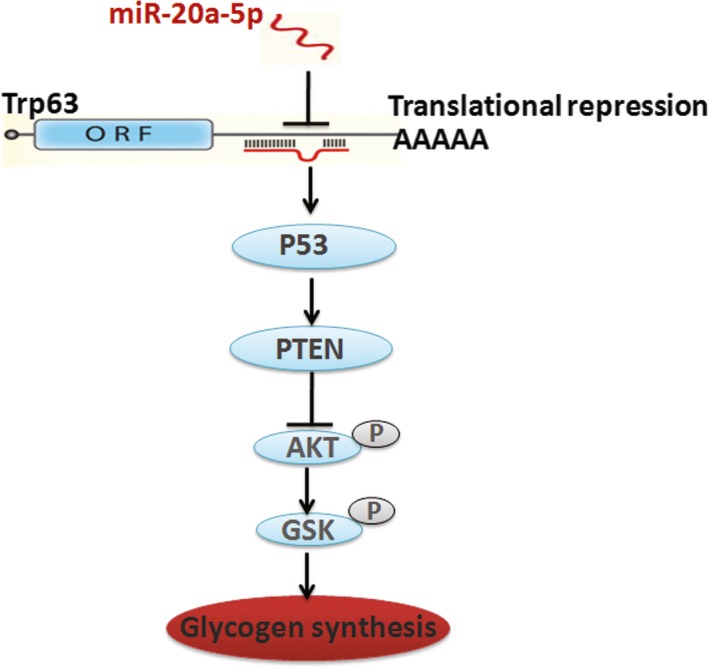
Proposed mechanisms by which miR‐20a‐5p contributes to glycogenesis in hepatocytes. MiR‐20a‐5p targets p63 to regulate p53 and PTEN expression, in turn contributes to glycogenesis in hepatocytes.

In summary, as shown in Figure 6, our findings provide mechanistic insight into the effects of miR‐20a‐5p on the regulation of AKT/GSK pathway and glycogenesis in hepatocytes. MiR‐20a‐5p might contribute to hepatic glycogen synthesis through targeting p63 to regulate p53 and PTEN expression. However, we need to further investigate the effects of miR‐20a‐5p overexpression in the liver of db/db mice on glucose metabolism.

## Conflicts of interest

The authors who have taken part in this study declared that they do not have anything to disclose regarding funding or conflict of interest with respect to this manuscript.

## References

[1] Zhong L , Zhu K , Jin N , *et al* A systematic analysis of miRNA‐mRNA paired variations reveals widespread miRNA misregulation in breast cancer. BioMed Res Int. 2014; 2014: 291280. 24949432 10.1155/2014/291280 PMC4052615

[2] Bartel DP . MicroRNAs: genomics, biogenesis, mechanism, and function. Cell. 2004; 116: 281–97. 14744438 10.1016/s0092-8674(04)00045-5

[3] Hansen TB , Jensen TI , Clausen BH , *et al* Natural RNA circles function as efficient microRNA sponges. Nature. 2013; 495: 384–8. 23446346 10.1038/nature11993

[4] He A , Zhu L , Gupta N , *et al* Overexpression of micro ribonucleic acid 29, highly up‐regulated in diabetic rats, leads to insulin resistance in 3T3‐L1 adipocytes. Mol Endocrinol. 2007; 21: 2785–94. 17652184 10.1210/me.2007-0167

[5] Trajkovski M , Hausser J , Soutschek J , *et al* MicroRNAs 103 and 107 regulate insulin sensitivity. Nature. 2011; 474: 649–53. 21654750 10.1038/nature10112

[6] Ling HY , Ou HS , Feng SD , *et al* CHANGES IN microRNA (miR) profile and effects of miR‐320 in insulin‐resistant 3T3‐L1 adipocytes. Clin Exp Pharmacol Physiol. 2009; 36: e32–9. 19473196 10.1111/j.1440-1681.2009.05207.x

[7] Ryu HS , Park SY , Ma D , *et al* The induction of microRNA targeting IRS‐1 is involved in the development of insulin resistance under conditions of mitochondrial dysfunction in hepatocytes. PLoS ONE. 2011; 6: e17343. 21464990 10.1371/journal.pone.0017343 PMC3064581

[8] Yang WM , Jeong HJ , Park SW , *et al* Obesity‐induced miR‐15b is linked causally to the development of insulin resistance through the repression of the insulin receptor in hepatocytes. Mol Nutr Food Res. 2015; 59: 2302–14. 10.1002/mnfr.201500107 26179126

[9] Dou L , Wang S , Sui X , *et al* MiR‐301a mediates the effect of IL‐6 on the AKT/GSK pathway and hepatic glycogenesis by regulating PTEN expression. Cell Physiol Biochem. 2015; 35: 1413–24. 25790935 10.1159/000373962

[10] Dou L , Zhao T , Wang L , *et al* miR‐200s contribute to interleukin‐6 (IL‐6)‐induced insulin resistance in hepatocytes. J Biol Chem. 2013; 288: 22596–606. 23798681 10.1074/jbc.M112.423145 PMC3829346

[11] Dou L , Meng X , Sui X , *et al* MiR‐19a regulates PTEN expression to mediate glycogen synthesis in hepatocytes. Sci Rep. 2015; 5: 11602. 26111969 10.1038/srep11602 PMC4481380

[12] Hossain A , Kuo MT , Saunders GF . Mir‐17‐5p regulates breast cancer cell proliferation by inhibiting translation of AIB1 mRNA. Mol Cell Biol. 2006; 26: 8191–201. 16940181 10.1128/MCB.00242-06 PMC1636750

[13] Yu Z , Wang C , Wang M , *et al* A cyclin D1/microRNA 17/20 regulatory feedback loop in control of breast cancer cell proliferation. J Cell Biol. 2008; 182: 509–17. 18695042 10.1083/jcb.200801079 PMC2500136

[14] Yu Z , Xu Z , Disante G , *et al* miR‐17/20 sensitization of breast cancer cells to chemotherapy‐induced apoptosis requires Akt1. Oncotarget. 2014; 5: 1083–90. 24658544 10.18632/oncotarget.1804 PMC4011585

[15] Guibinga GH , Murray F , Barron N , *et al* Deficiency of the purine metabolic gene HPRT dysregulates microRNA‐17 family cluster and guanine‐based cellular functions: a role for EPAC in Lesch‐Nyhan syndrome. Hum Mol Genet. 2013; 22: 4502–15. 23804752 10.1093/hmg/ddt298 PMC3888132

[16] Jiang Z , Yin J , Fu W , *et al* MiRNA 17 family regulates cisplatin‐resistant and metastasis by targeting TGFbetaR2 in NSCLC. PLoS ONE. 2014; 9: e94639. 24722426 10.1371/journal.pone.0094639 PMC3983236

[17] Meira M , Sievers C , Hoffmann F , *et al* Unraveling natalizumab effects on deregulated miR‐17 expression in CD4^+^ T cells of patients with relapsing‐remitting multiple sclerosis. J Immunol Res. 2014; 2014: 897249. 24901013 10.1155/2014/897249 PMC4036714

[18] Vasilatou D , Papageorgiou SG , Kontsioti F , *et al* Expression analysis of mir‐17‐5p, mir‐20a and let‐7a microRNAs and their target proteins in CD34^+^ bone marrow cells of patients with myelodysplastic syndromes. Leuk Res. 2013; 37: 251–8. 23246221 10.1016/j.leukres.2012.11.011

[19] Li AG , Piluso LG , Cai X , *et al* Mechanistic insights into maintenance of high p53 acetylation by PTEN. Mol Cell. 2006; 23: 575–87. 16916644 10.1016/j.molcel.2006.06.028

[20] Zhi F , Shao N , Wang R , *et al* Identification of 9 serum microRNAs as potential noninvasive biomarkers of human astrocytoma. Neuro Oncol. 2015; 17: 383–91. 25140035 10.1093/neuonc/nou169 PMC4483096

[21] Rezaei M , Andrieu T , Neuenschwander S , *et al* Regulation of 11beta‐hydroxysteroid dehydrogenase type 2 by microRNA. Hypertension. 2014; 64: 860–6. 24980668 10.1161/HYPERTENSIONAHA.114.00002

[22] Wen Y , Han J , Chen J , *et al* Plasma miRNAs as early biomarkers for detecting hepatocellular carcinoma. Int J Cancer. 2015; 137: 1679–90. 25845839 10.1002/ijc.29544

[23] Zhu Y , Tian F , Li H , *et al* Profiling maternal plasma microRNA expression in early pregnancy to predict gestational diabetes mellitus. Int J Gynaecol Obstet. 2015; 130: 49–53. 25887942 10.1016/j.ijgo.2015.01.010

[24] Angerstein C , Hecker M , Paap BK , *et al* Integration of MicroRNA databases to study MicroRNAs associated with multiple sclerosis. Mol Neurobiol. 2012; 45: 520–35. 22549745 10.1007/s12035-012-8270-0

[25] Ye SB , Li ZL , Luo DH , *et al* Tumor‐derived exosomes promote tumor progression and T‐cell dysfunction through the regulation of enriched exosomal microRNAs in human nasopharyngeal carcinoma. Oncotarget. 2014; 5: 5439–52. 24978137 10.18632/oncotarget.2118 PMC4170615

[26] Wang IK , Sun KT , Tsai TH , *et al* MiR‐20a‐5p mediates hypoxia‐induced autophagy by targeting ATG16L1 in ischemic kidney injury. Life Sci. 2015; 136: 133–141. 26165754 10.1016/j.lfs.2015.07.002

[27] Xu Q , Dong QG , Sun LP , *et al* Expression of serum miR‐20a‐5p, let‐7a, and miR‐320a and their correlations with pepsinogen in atrophic gastritis and gastric cancer: a case‐control study. BMC Clin Pathol. 2013; 13: 11. 23521833 10.1186/1472-6890-13-11 PMC3635921

[28] Zhang JX , Song W , Chen ZH , *et al* Prognostic and predictive value of a microRNA signature in stage II colon cancer: a microRNA expression analysis. Lancet Oncol. 2013; 14: 1295–306. 24239208 10.1016/S1470-2045(13)70491-1

[29] Calvano Filho CM , Calvano‐Mendes DC , Carvalho KC , *et al* Triple‐negative and luminal A breast tumors: differential expression of miR‐18a‐5p, miR‐17‐5p, and miR‐20a‐5p. Tumour Biol. 2014; 35: 7733–41. 24810926 10.1007/s13277-014-2025-7

[30] Yao JY , Chen JK . Roles of p63 in epidermal development and tumorigenesis. Biomed J. 2012; 35: 457–63. 23442358 10.4103/2319-4170.104410

[31] Bir F , Aksoy Altinboga A , Satiroglu Tufan NL , *et al* Potential utility of p63 expression in differential diagnosis of non‐small‐cell lung carcinoma and its effect on prognosis of the disease. Med Sci Monit. 2014; 20: 219–26. 24509874 10.12659/MSM.890394 PMC3930580

[32] Leao M , Gomes S , Bessa C , *et al* Studying p53 family proteins in yeast: induction of autophagic cell death and modulation by interactors and small molecules. Exp Cell Res. 2015; 330: 164–77. 25265062 10.1016/j.yexcr.2014.09.028

[33] Ng WL , Chen G , Wang M , *et al* OCT4 as a target of miR‐34a stimulates p63 but inhibits p53 to promote human cell transformation. Cell Death Dis. 2014; 5: e1024. 24457968 10.1038/cddis.2013.563 PMC4040665

[34] Vandormael‐Pournin S , Guigon CJ , Ishaq M , *et al* Oocyte‐specific inactivation of Omcg1 leads to DNA damage and c‐Abl/TAp63‐dependent oocyte death associated with dramatic remodeling of ovarian somatic cells. Cell Death Differ. 2015; 22: 108–17. 25168238 10.1038/cdd.2014.122 PMC4262775

[35] Masse I , Barbollat‐Boutrand L , Molina M , *et al* Functional interplay between p63 and p53 controls RUNX1 function in the transition from proliferation to differentiation in human keratinocytes. Cell Death Dis. 2012; 3: e318. 22673192 10.1038/cddis.2012.62 PMC3388234

[36] Fatt MP , Cancino GI , Miller FD , *et al* p63 and p73 coordinate p53 function to determine the balance between survival, cell death, and senescence in adult neural precursor cells. Cell Death Differ. 2014; 21: 1546–59. 24809925 10.1038/cdd.2014.61 PMC4158681

[37] Urist MJ , Di Como CJ , Lu ML , *et al* Loss of p63 expression is associated with tumor progression in bladder cancer. Am J Pathol. 2002; 161: 1199–206. 12368193 10.1016/S0002-9440(10)64396-9 PMC1867279

[38] Koga F , Kawakami S , Fujii Y , *et al* Impaired p63 expression associates with poor prognosis and uroplakin III expression in invasive urothelial carcinoma of the bladder. Clin Cancer Res. 2003; 9: 5501–7. 14654529

[39] Muller PA , Caswell PT , Doyle B , *et al* Mutant p53 drives invasion by promoting integrin recycling. Cell. 2009; 139: 1327–41. 20064378 10.1016/j.cell.2009.11.026

[40] Adorno M , Cordenonsi M , Montagner M , *et al* A Mutant‐p53/Smad complex opposes p63 to empower TGFbeta‐induced metastasis. Cell. 2009; 137: 87–98. 19345189 10.1016/j.cell.2009.01.039

[41] Su X , Chakravarti D , Cho MS , *et al* TAp63 suppresses metastasis through coordinate regulation of Dicer and miRNAs. Nature. 2010; 467: 986–90. 20962848 10.1038/nature09459 PMC3055799

[42] Li Y , Prives C . Are interactions with p63 and p73 involved in mutant p53 gain of oncogenic function? Oncogene. 2007; 26: 2220–5. 17401431 10.1038/sj.onc.1210311

[43] Keyes WM , Wu Y , Vogel H , *et al* p63 deficiency activates a program of cellular senescence and leads to accelerated aging. Genes Dev. 2005; 19: 1986–99. 16107615 10.1101/gad.342305 PMC1199570

[44] Arcolino FO , Ribeiro DL , Gobbo MG , *et al* Proliferation and apoptotic rates and increased frequency of p63‐positive cells in the prostate acinar epithelium of alloxan‐induced diabetic rats. Int J Exp Pathol. 2010; 91: 144–54. 20041964 10.1111/j.1365-2613.2009.00696.x PMC2965900

[45] Zhao K , Zhou Y , Qiao C , *et al* Oroxylin A promotes PTEN‐mediated negative regulation of MDM2 transcription *via* SIRT3‐mediated deacetylation to stabilize p53 and inhibit glycolysis in wt‐p53 cancer cells. J Hematol Oncol. 2015; 8: 41. 25902914 10.1186/s13045-015-0137-1 PMC4419472

[46] Stambolic V , MacPherson D , Sas D , *et al* Regulation of PTEN transcription by p53. Mol Cell. 2001; 8: 317–25. 11545734 10.1016/s1097-2765(01)00323-9

[47] Chen Z , Trotman LC , Shaffer D , *et al* Crucial role of p53‐dependent cellular senescence in suppression of Pten‐deficient tumorigenesis. Nature. 2005; 436: 725–30. 16079851 10.1038/nature03918 PMC1939938

[48] Nakanishi A , Kitagishi Y , Ogura Y , *et al* The tumor suppressor PTEN interacts with p53 in hereditary cancer (Review). Int J Oncol. 2014; 44: 1813–9. 24718924 10.3892/ijo.2014.2377

[49] Freeman DJ , Li AG , Wei G , *et al* PTEN tumor suppressor regulates p53 protein levels and activity through phosphatase‐dependent and ‐independent mechanisms. Cancer Cell. 2003; 3: 117–30. 12620407 10.1016/s1535-6108(03)00021-7

[50] Puszynski K , Hat B , Lipniacki T . Oscillations and bistability in the stochastic model of p53 regulation. J Theor Biol. 2008; 254: 452–65. 18577387 10.1016/j.jtbi.2008.05.039

[51] Zhou M , Gu L , Findley HW , *et al* PTEN reverses MDM2‐mediated chemotherapy resistance by interacting with p53 in acute lymphoblastic leukemia cells. Cancer Res. 2003; 63: 6357–62. 14559824

